# 2-[5-(4-Meth­oxy­phen­yl)-3-phenyl-4,5-dihydro-1*H*-pyrazol-1-yl]-6-methyl-1,3-benzothia­zole

**DOI:** 10.1107/S1600536811033666

**Published:** 2011-08-27

**Authors:** Hoong-Kun Fun, Suhana Arshad, M. Himaja, D. Munirajasekhar, B. K. Sarojini

**Affiliations:** a X-ray Crystallography Unit, School of Physics, Universiti Sains Malaysia, 11800 USM, Penang, Malaysia; b Chemistry Division, School of Advanced Sciences, VIT University, Vellore 632014, Tamil Nadu, India; c Department of Chemistry, P. A. College of Engineering, Nadupadavu, D. K., Mangalore, India

## Abstract

In the title compound, C_24_H_21_N_3_OS, the pyrazole ring makes dihedral angles of 5.40 (7) and 6.72 (8)° with the benzo[*d*]thiazole ring system and the benzene ring, respectively, and a dihedral angle of 85.72 (8)° with the meth­oxy-substituted benzene ring. In the crystal structure, the mol­ecules are linked by C—H⋯π inter­actions.

## Related literature

For background to the properties and applications of pyrazolines, see: Taylor *et al.* (1992 ▶); Rajendera Prasad *et al.* (2005 ▶). For reference bond-length data, see: Allen *et al.* (1987 ▶).
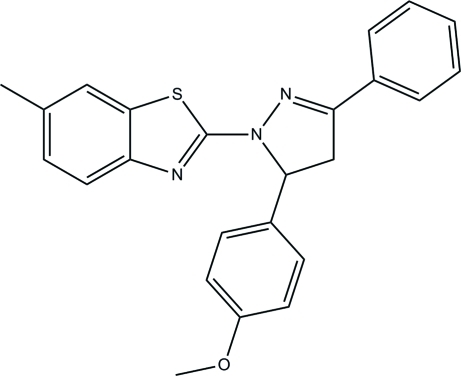


## Experimental

### 

#### Crystal data

C_24_H_21_N_3_OS
*M* *_r_* = 399.50
Orthorhombic, 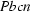

*a* = 22.632 (3) Å
*b* = 11.1961 (12) Å
*c* = 16.1137 (18) Å
*V* = 4083.1 (8) Å^3^
*Z* = 8
Mo *K*α radiation
μ = 0.18 mm^−1^
*T* = 296 K
0.37 × 0.24 × 0.19 mm

#### Data collection

Bruker SMART APEXII DUO CCD diffractometer
Absorption correction: multi-scan (*SADABS*; Bruker, 2009 ▶) *T*_min_ = 0.936, *T*_max_ = 0.967
25081 measured reflections
5949 independent reflections
3835 reflections with *I* > 2σ(*I*)
*R*_int_ = 0.041

#### Refinement

*R*[*F* ^2^ > 2σ(*F* ^2^)] = 0.041
*wR*(*F* ^2^) = 0.123
*S* = 1.00
5949 reflections
264 parameters
H-atom parameters constrained
Δρ_max_ = 0.25 e Å^−3^
Δρ_min_ = −0.29 e Å^−3^

### 

Data collection: *APEX2* (Bruker, 2009 ▶); cell refinement: *SAINT* (Bruker, 2009 ▶); data reduction: *SAINT*; program(s) used to solve structure: *SHELXTL* (Sheldrick, 2008 ▶); program(s) used to refine structure: *SHELXTL*; molecular graphics: *SHELXTL*; software used to prepare material for publication: *SHELXTL* and *PLATON* (Spek, 2009 ▶).

## Supplementary Material

Crystal structure: contains datablock(s) global, I. DOI: 10.1107/S1600536811033666/hb6368sup1.cif

Structure factors: contains datablock(s) I. DOI: 10.1107/S1600536811033666/hb6368Isup2.hkl

Supplementary material file. DOI: 10.1107/S1600536811033666/hb6368Isup3.cml

Additional supplementary materials:  crystallographic information; 3D view; checkCIF report

## Figures and Tables

**Table 1:** Hydrogen-bond geometry (Å, °) *Cg*1, *Cg*2 and *Cg*3 are the centroids of the S1/C17/N1/C18/C23, C1–C6 and C10–C15 rings, respectively.

*D*—H⋯*A*	*D*—H	H⋯*A*	*D*⋯*A*	*D*—H⋯*A*
C15—H15*A*⋯*Cg*1^i^	0.93	2.91	3.6318 (17)	138
C22—H22*A*⋯*Cg*2^i^	0.93	2.89	3.6438 (18)	140
C2—H2*A*⋯*Cg*3^ii^	0.93	2.74	3.4884 (18)	138

Symmetry codes: (i) 
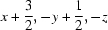
; (ii) 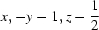.

**Fig. 1.**
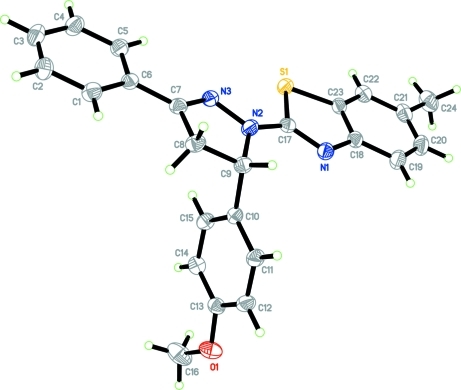
The molecular structure of the title compound, showing 30% probability displacement ellipsoids.

**Fig. 2.**
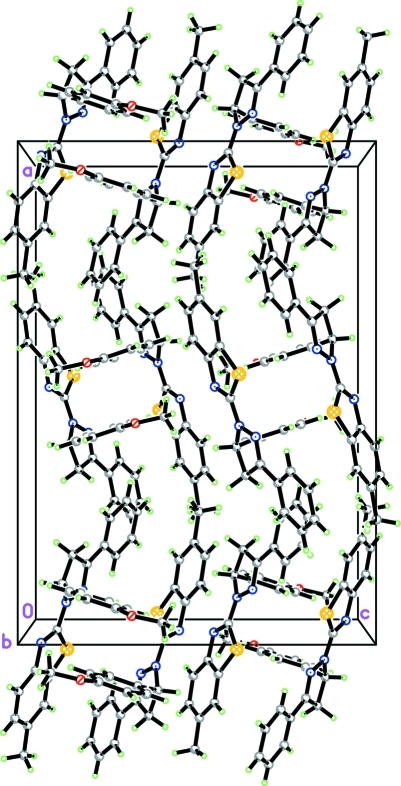
The crystal packing of the title compound.

## supplementary crystallographic information

### Comment

Pyrazolines are an important class of heterocyclic compounds, some of which exhibit important pharmacological activities such as antitumor (Taylor *et al.*, 1992) and antidepressant (Rajendera Prasad *et al.*, 2005) agents. The title compound, (I), was synthesized by the condensation of 1-(6-methylbenzo[*d*]thiazol-2-yl)hydrazine with (*E*)-3-(4-methoxyphenyl)-1-phenylprop-2-en-1-one in presence of ethanol and its crystal structure is now described.

In the molecular structure (Fig 1), the pyrazole ring (N2/N3/C7–C9) is approximately planar with the benzo[*d*]thiozole ring system (S1/N1/C17–C23) and the benzene ring (C1–C6) with dihedral angles of 5.40 (7)° and 6.72 (8)°, respectively. On the other hand, the pyrazole ring (N2/N3/C7–C9) is approximately perpendicular to the methoxy substituted benzene ring (C10–C15) with dihedral angle of 85.72 (8)°. The bond lengths (Allen *et al.*, 1987) and angles are within normal ranges.

The crystal packing is shown in Fig. 2. The crystal structure is stabilized by the intermolecular C15–H15A···*Cg*1, C22—H22A···*Cg*2 and C2—H2A···*Cg*3 (Table 1) interactions (*Cg*1, *Cg*2 and *Cg*3 are the centroids of S1/C17/N1/C18/C23, C1—C6 and C10—C15 rings, respectively).

### Experimental

A mixture of (*E*)-3-(4-methoxyphenyl)-1-phenylprop-2-en-1-one (5 mmol) and 1-(6-methylbenzo[*d*]thiazol-2-yl)hydrazine (5 mmol) was refluxed for 16 h in ethanol (20 ml). After completion of the reaction, the reaction mixture was poured into cold water. The precipitate obtained was filtered and washed with cold water. The product was recrystalized from ethanol to yield colourless blocks of (I) and dried. m.p. 131–132 °C, HRMS Calcd for C_24_H_21_N_3_OS 399.5080 found 399.5079.

### Refinement

All H atoms were positioned geometrically [C—H = 0.93–0.98 Å] and refined using a riding model with *U*_iso_(H) = 1.2 or 1.5 *U*_eq_(C). A rotating group model was applied to the methyl groups.

### Crystal data

**Table:**

C_24_H_21_N_3_OS	*F*(000) = 1680
*M**_r_* = 399.50	*D*_x_ = 1.300 Mg m^−^^3^
Orthorhombic, *P**b**c**n*	Mo *K*α radiation, λ = 0.71073 Å
Hall symbol: -P 2n 2ab	Cell parameters from 4472 reflections
*a* = 22.632 (3) Å	θ = 2.4–27.1°
*b* = 11.1961 (12) Å	µ = 0.18 mm^−^^1^
*c* = 16.1137 (18) Å	*T* = 296 K
*V* = 4083.1 (8) Å^3^	Block, colourless
*Z* = 8	0.37 × 0.24 × 0.19 mm

### Data collection

**Table:**

Bruker SMART APEXII DUO CCD diffractometer	5949 independent reflections
Radiation source: fine-focus sealed tube	3835 reflections with *I* > 2σ(*I*)
graphite	*R*_int_ = 0.041
φ and ω scans	θ_max_ = 30.0°, θ_min_ = 1.8°
Absorption correction: multi-scan (*SADABS*; Bruker, 2009)	*h* = −31→31
*T*_min_ = 0.936, *T*_max_ = 0.967	*k* = −15→10
25081 measured reflections	*l* = −22→18

### Refinement

**Table:**

Refinement on *F*^2^	Primary atom site location: structure-invariant direct methods
Least-squares matrix: full	Secondary atom site location: difference Fourier map
*R*[*F*^2^ > 2σ(*F*^2^)] = 0.041	Hydrogen site location: inferred from neighbouring sites
*wR*(*F*^2^) = 0.123	H-atom parameters constrained
*S* = 1.00	*w* = 1/[σ^2^(*F*_o_^2^) + (0.0593*P*)^2^ + 0.3403*P*] where *P* = (*F*_o_^2^ + 2*F*_c_^2^)/3
5949 reflections	(Δ/σ)_max_ = 0.002
264 parameters	Δρ_max_ = 0.25 e Å^−^^3^
0 restraints	Δρ_min_ = −0.29 e Å^−^^3^

### Special details

**Table:**

Geometry. All e.s.d.'s (except the e.s.d. in the dihedral angle between two l.s. planes) are estimated using the full covariance matrix. The cell e.s.d.'s are taken into account individually in the estimation of e.s.d.'s in distances, angles and torsion angles; correlations between e.s.d.'s in cell parameters are only used when they are defined by crystal symmetry. An approximate (isotropic) treatment of cell e.s.d.'s is used for estimating e.s.d.'s involving l.s. planes.
Refinement. Refinement of *F*^2^ against ALL reflections. The weighted *R*-factor *wR* and goodness of fit *S* are based on *F*^2^, conventional *R*-factors *R* are based on *F*, with *F* set to zero for negative *F*^2^. The threshold expression of *F*^2^ > σ(*F*^2^) is used only for calculating *R*-factors(gt) *etc*. and is not relevant to the choice of reflections for refinement. *R*-factors based on *F*^2^ are statistically about twice as large as those based on *F*, and *R*-factors based on ALL data will be even larger.

### Fractional atomic coordinates and isotropic or equivalent isotropic displacement parameters (Å^2^)

**Table:**

	*x*	*y*	*z*	*U*_iso_*/*U*_eq_	
S1	0.537594 (17)	−0.00428 (3)	0.11748 (3)	0.04063 (11)	
N1	0.51267 (5)	0.20397 (10)	0.05137 (8)	0.0394 (3)	
N2	0.43376 (5)	0.11316 (10)	0.11985 (8)	0.0408 (3)	
N3	0.41278 (5)	0.01631 (10)	0.16416 (8)	0.0382 (3)	
O1	0.43721 (6)	0.61031 (10)	0.32050 (8)	0.0589 (3)	
C1	0.26306 (7)	−0.02592 (14)	0.24336 (11)	0.0463 (4)	
H1A	0.2459	0.0437	0.2231	0.056*	
C2	0.22952 (7)	−0.10444 (16)	0.29063 (11)	0.0529 (4)	
H2A	0.1902	−0.0867	0.3023	0.063*	
C3	0.25414 (8)	−0.20806 (16)	0.32013 (11)	0.0537 (4)	
H3A	0.2315	−0.2608	0.3514	0.064*	
C4	0.31269 (8)	−0.23390 (15)	0.30326 (11)	0.0535 (4)	
H4A	0.3293	−0.3041	0.3235	0.064*	
C5	0.34660 (7)	−0.15656 (13)	0.25674 (10)	0.0450 (4)	
H5A	0.3859	−0.1748	0.2456	0.054*	
C6	0.32201 (6)	−0.05056 (12)	0.22615 (9)	0.0378 (3)	
C7	0.35724 (6)	0.03383 (12)	0.17774 (10)	0.0377 (3)	
C8	0.33397 (7)	0.14714 (13)	0.13926 (10)	0.0427 (4)	
H8A	0.3117	0.1938	0.1792	0.051*	
H8B	0.3091	0.1301	0.0917	0.051*	
C9	0.39093 (6)	0.21231 (12)	0.11292 (10)	0.0384 (3)	
H9A	0.3879	0.2390	0.0552	0.046*	
C10	0.40585 (6)	0.31648 (12)	0.16894 (9)	0.0340 (3)	
C11	0.39769 (7)	0.43310 (13)	0.14186 (10)	0.0423 (4)	
H11A	0.3851	0.4470	0.0878	0.051*	
C12	0.40797 (8)	0.52875 (14)	0.19391 (10)	0.0471 (4)	
H12A	0.4020	0.6062	0.1749	0.056*	
C13	0.42714 (6)	0.50954 (13)	0.27438 (10)	0.0396 (3)	
C14	0.43514 (7)	0.39440 (13)	0.30260 (10)	0.0411 (3)	
H14A	0.4477	0.3807	0.3567	0.049*	
C15	0.42441 (7)	0.29915 (13)	0.24975 (10)	0.0399 (3)	
H15A	0.4298	0.2217	0.2691	0.048*	
C16	0.45090 (10)	0.5966 (2)	0.40532 (13)	0.0768 (6)	
H16A	0.4569	0.6737	0.4299	0.115*	
H16B	0.4863	0.5499	0.4109	0.115*	
H16C	0.4189	0.5567	0.4329	0.115*	
C17	0.49059 (6)	0.11551 (12)	0.09394 (9)	0.0348 (3)	
C18	0.57140 (6)	0.17917 (13)	0.03344 (9)	0.0388 (3)	
C19	0.60917 (8)	0.25405 (16)	−0.01089 (10)	0.0508 (4)	
H19A	0.5956	0.3265	−0.0317	0.061*	
C20	0.66700 (8)	0.21907 (17)	−0.02351 (11)	0.0550 (4)	
H20A	0.6919	0.2688	−0.0538	0.066*	
C21	0.68940 (7)	0.11232 (16)	0.00738 (10)	0.0498 (4)	
C22	0.65199 (7)	0.03749 (15)	0.05197 (10)	0.0455 (4)	
H22A	0.6660	−0.0343	0.0734	0.055*	
C23	0.59353 (6)	0.07106 (13)	0.06414 (9)	0.0375 (3)	
C24	0.75341 (8)	0.0795 (2)	−0.00596 (13)	0.0688 (5)	
H24A	0.7616	0.0046	0.0207	0.103*	
H24B	0.7782	0.1405	0.0173	0.103*	
H24C	0.7611	0.0727	−0.0644	0.103*	

### Atomic displacement parameters (Å^2^)

**Table:**

	*U*^11^	*U*^22^	*U*^33^	*U*^12^	*U*^13^	*U*^23^
S1	0.0411 (2)	0.03568 (19)	0.0451 (2)	−0.00003 (15)	0.00495 (16)	0.00259 (15)
N1	0.0411 (7)	0.0400 (7)	0.0372 (7)	−0.0024 (5)	0.0005 (5)	0.0015 (5)
N2	0.0386 (7)	0.0320 (6)	0.0519 (8)	−0.0002 (5)	0.0072 (6)	0.0028 (5)
N3	0.0376 (6)	0.0334 (6)	0.0435 (8)	−0.0027 (5)	0.0044 (5)	−0.0023 (5)
O1	0.0692 (8)	0.0499 (7)	0.0575 (8)	0.0019 (6)	−0.0072 (6)	−0.0192 (6)
C1	0.0370 (7)	0.0434 (8)	0.0585 (11)	−0.0011 (6)	−0.0023 (7)	−0.0003 (7)
C2	0.0336 (8)	0.0619 (11)	0.0631 (12)	−0.0049 (7)	0.0055 (7)	−0.0002 (9)
C3	0.0483 (9)	0.0560 (10)	0.0568 (11)	−0.0135 (8)	0.0059 (8)	0.0069 (8)
C4	0.0527 (10)	0.0435 (9)	0.0642 (12)	−0.0015 (7)	0.0048 (8)	0.0091 (8)
C5	0.0399 (8)	0.0400 (8)	0.0551 (10)	0.0008 (6)	0.0041 (7)	−0.0010 (7)
C6	0.0353 (7)	0.0369 (7)	0.0411 (9)	−0.0048 (6)	−0.0001 (6)	−0.0053 (6)
C7	0.0359 (7)	0.0348 (7)	0.0423 (9)	−0.0030 (6)	−0.0017 (6)	−0.0057 (6)
C8	0.0356 (7)	0.0377 (8)	0.0548 (10)	−0.0020 (6)	−0.0040 (7)	−0.0013 (7)
C9	0.0388 (7)	0.0360 (7)	0.0405 (8)	0.0002 (6)	−0.0031 (6)	0.0009 (6)
C10	0.0311 (7)	0.0339 (7)	0.0369 (8)	0.0001 (5)	−0.0003 (6)	0.0027 (6)
C11	0.0536 (9)	0.0375 (8)	0.0358 (8)	0.0010 (6)	−0.0030 (7)	0.0059 (6)
C12	0.0623 (10)	0.0324 (7)	0.0465 (10)	0.0029 (7)	0.0002 (8)	0.0036 (6)
C13	0.0337 (7)	0.0419 (8)	0.0434 (9)	0.0010 (6)	0.0013 (6)	−0.0056 (7)
C14	0.0370 (7)	0.0487 (8)	0.0374 (8)	0.0031 (6)	−0.0052 (6)	0.0008 (6)
C15	0.0399 (8)	0.0372 (7)	0.0425 (9)	0.0025 (6)	−0.0052 (6)	0.0062 (6)
C16	0.0759 (14)	0.0946 (16)	0.0600 (13)	0.0059 (12)	−0.0087 (11)	−0.0341 (12)
C17	0.0376 (7)	0.0346 (7)	0.0322 (8)	−0.0022 (6)	−0.0009 (6)	−0.0048 (6)
C18	0.0409 (8)	0.0436 (8)	0.0319 (8)	−0.0050 (6)	0.0007 (6)	−0.0028 (6)
C19	0.0548 (10)	0.0510 (10)	0.0466 (10)	−0.0072 (7)	0.0057 (8)	0.0075 (8)
C20	0.0513 (10)	0.0659 (11)	0.0477 (10)	−0.0166 (8)	0.0107 (8)	0.0010 (8)
C21	0.0401 (8)	0.0671 (11)	0.0422 (9)	−0.0069 (8)	0.0043 (7)	−0.0114 (8)
C22	0.0420 (8)	0.0516 (9)	0.0429 (9)	0.0011 (7)	0.0006 (7)	−0.0048 (7)
C23	0.0399 (7)	0.0413 (8)	0.0314 (8)	−0.0042 (6)	0.0021 (6)	−0.0019 (6)
C24	0.0437 (9)	0.0941 (15)	0.0686 (13)	−0.0049 (10)	0.0110 (9)	−0.0166 (11)

### Geometric parameters (Å, °)

**Table:**

S1—C23	1.7473 (15)	C9—H9A	0.9800
S1—C17	1.7534 (15)	C10—C15	1.382 (2)
N1—C17	1.3042 (18)	C10—C11	1.3891 (19)
N1—C18	1.3884 (19)	C11—C12	1.380 (2)
N2—C17	1.3525 (18)	C11—H11A	0.9300
N2—N3	1.3824 (16)	C12—C13	1.384 (2)
N2—C9	1.4780 (18)	C12—H12A	0.9300
N3—C7	1.2909 (18)	C13—C14	1.379 (2)
O1—C13	1.3701 (17)	C14—C15	1.386 (2)
O1—C16	1.410 (2)	C14—H14A	0.9300
C1—C2	1.389 (2)	C15—H15A	0.9300
C1—C6	1.390 (2)	C16—H16A	0.9600
C1—H1A	0.9300	C16—H16B	0.9600
C2—C3	1.372 (2)	C16—H16C	0.9600
C2—H2A	0.9300	C18—C19	1.394 (2)
C3—C4	1.383 (2)	C18—C23	1.400 (2)
C3—H3A	0.9300	C19—C20	1.381 (2)
C4—C5	1.379 (2)	C19—H19A	0.9300
C4—H4A	0.9300	C20—C21	1.390 (3)
C5—C6	1.400 (2)	C20—H20A	0.9300
C5—H5A	0.9300	C21—C22	1.391 (2)
C6—C7	1.462 (2)	C21—C24	1.510 (2)
C7—C8	1.507 (2)	C22—C23	1.389 (2)
C8—C9	1.541 (2)	C22—H22A	0.9300
C8—H8A	0.9700	C24—H24A	0.9600
C8—H8B	0.9700	C24—H24B	0.9600
C9—C10	1.513 (2)	C24—H24C	0.9600
			
C23—S1—C17	87.93 (7)	C11—C12—C13	120.11 (14)
C17—N1—C18	108.92 (12)	C11—C12—H12A	119.9
C17—N2—N3	120.09 (11)	C13—C12—H12A	119.9
C17—N2—C9	125.85 (12)	O1—C13—C14	124.69 (14)
N3—N2—C9	113.76 (11)	O1—C13—C12	115.62 (13)
C7—N3—N2	107.63 (12)	C14—C13—C12	119.69 (13)
C13—O1—C16	118.20 (14)	C13—C14—C15	119.58 (14)
C2—C1—C6	120.55 (15)	C13—C14—H14A	120.2
C2—C1—H1A	119.7	C15—C14—H14A	120.2
C6—C1—H1A	119.7	C10—C15—C14	121.61 (13)
C3—C2—C1	120.21 (15)	C10—C15—H15A	119.2
C3—C2—H2A	119.9	C14—C15—H15A	119.2
C1—C2—H2A	119.9	O1—C16—H16A	109.5
C2—C3—C4	119.85 (15)	O1—C16—H16B	109.5
C2—C3—H3A	120.1	H16A—C16—H16B	109.5
C4—C3—H3A	120.1	O1—C16—H16C	109.5
C5—C4—C3	120.58 (16)	H16A—C16—H16C	109.5
C5—C4—H4A	119.7	H16B—C16—H16C	109.5
C3—C4—H4A	119.7	N1—C17—N2	122.78 (13)
C4—C5—C6	120.16 (15)	N1—C17—S1	117.53 (11)
C4—C5—H5A	119.9	N2—C17—S1	119.69 (10)
C6—C5—H5A	119.9	N1—C18—C19	124.98 (14)
C1—C6—C5	118.64 (14)	N1—C18—C23	116.23 (13)
C1—C6—C7	120.11 (14)	C19—C18—C23	118.78 (14)
C5—C6—C7	121.25 (13)	C20—C19—C18	119.07 (16)
N3—C7—C6	121.56 (13)	C20—C19—H19A	120.5
N3—C7—C8	113.48 (13)	C18—C19—H19A	120.5
C6—C7—C8	124.96 (13)	C19—C20—C21	122.44 (15)
C7—C8—C9	102.69 (12)	C19—C20—H20A	118.8
C7—C8—H8A	111.2	C21—C20—H20A	118.8
C9—C8—H8A	111.2	C20—C21—C22	118.73 (15)
C7—C8—H8B	111.2	C20—C21—C24	120.51 (16)
C9—C8—H8B	111.2	C22—C21—C24	120.74 (17)
H8A—C8—H8B	109.1	C23—C22—C21	119.32 (16)
N2—C9—C10	112.80 (12)	C23—C22—H22A	120.3
N2—C9—C8	99.91 (11)	C21—C22—H22A	120.3
C10—C9—C8	112.79 (12)	C22—C23—C18	121.65 (14)
N2—C9—H9A	110.3	C22—C23—S1	128.96 (12)
C10—C9—H9A	110.3	C18—C23—S1	109.38 (11)
C8—C9—H9A	110.3	C21—C24—H24A	109.5
C15—C10—C11	117.93 (13)	C21—C24—H24B	109.5
C15—C10—C9	121.45 (12)	H24A—C24—H24B	109.5
C11—C10—C9	120.50 (13)	C21—C24—H24C	109.5
C12—C11—C10	121.07 (15)	H24A—C24—H24C	109.5
C12—C11—H11A	119.5	H24B—C24—H24C	109.5
C10—C11—H11A	119.5		
			
C17—N2—N3—C7	−177.07 (13)	C11—C12—C13—O1	178.73 (15)
C9—N2—N3—C7	8.82 (17)	C11—C12—C13—C14	−0.9 (2)
C6—C1—C2—C3	−0.7 (3)	O1—C13—C14—C15	−178.95 (14)
C1—C2—C3—C4	0.5 (3)	C12—C13—C14—C15	0.6 (2)
C2—C3—C4—C5	−0.2 (3)	C11—C10—C15—C14	−0.5 (2)
C3—C4—C5—C6	0.1 (3)	C9—C10—C15—C14	−176.56 (13)
C2—C1—C6—C5	0.6 (2)	C13—C14—C15—C10	0.1 (2)
C2—C1—C6—C7	−178.78 (15)	C18—N1—C17—N2	−179.24 (13)
C4—C5—C6—C1	−0.3 (2)	C18—N1—C17—S1	0.64 (16)
C4—C5—C6—C7	179.05 (15)	N3—N2—C17—N1	179.42 (13)
N2—N3—C7—C6	−177.70 (13)	C9—N2—C17—N1	−7.2 (2)
N2—N3—C7—C8	2.31 (17)	N3—N2—C17—S1	−0.46 (18)
C1—C6—C7—N3	176.59 (15)	C9—N2—C17—S1	172.88 (11)
C5—C6—C7—N3	−2.8 (2)	C23—S1—C17—N1	−0.27 (12)
C1—C6—C7—C8	−3.4 (2)	C23—S1—C17—N2	179.61 (12)
C5—C6—C7—C8	177.19 (14)	C17—N1—C18—C19	−179.67 (15)
N3—C7—C8—C9	−11.49 (17)	C17—N1—C18—C23	−0.79 (18)
C6—C7—C8—C9	168.52 (13)	N1—C18—C19—C20	179.20 (15)
C17—N2—C9—C10	−68.81 (19)	C23—C18—C19—C20	0.3 (2)
N3—N2—C9—C10	104.90 (14)	C18—C19—C20—C21	−0.9 (3)
C17—N2—C9—C8	171.19 (14)	C19—C20—C21—C22	0.6 (3)
N3—N2—C9—C8	−15.11 (16)	C19—C20—C21—C24	−178.27 (17)
C7—C8—C9—N2	14.51 (15)	C20—C21—C22—C23	0.2 (2)
C7—C8—C9—C10	−105.50 (14)	C24—C21—C22—C23	179.10 (15)
N2—C9—C10—C15	−42.67 (19)	C21—C22—C23—C18	−0.8 (2)
C8—C9—C10—C15	69.62 (17)	C21—C22—C23—S1	−179.64 (12)
N2—C9—C10—C11	141.35 (14)	N1—C18—C23—C22	−178.48 (13)
C8—C9—C10—C11	−106.37 (16)	C19—C18—C23—C22	0.5 (2)
C15—C10—C11—C12	0.2 (2)	N1—C18—C23—S1	0.60 (16)
C9—C10—C11—C12	176.33 (14)	C19—C18—C23—S1	179.55 (12)
C10—C11—C12—C13	0.5 (2)	C17—S1—C23—C22	178.81 (15)
C16—O1—C13—C14	−6.9 (2)	C17—S1—C23—C18	−0.18 (11)
C16—O1—C13—C12	173.59 (16)		

### Hydrogen-bond geometry (Å, °)

**Table:**

Cg1, Cg2 and Cg3 are the centroids of the S1/C17/N1/C18/C23, C1–C6 and C10–C15 rings, respectively.

**Table:**

*D*—H···*A*	*D*—H	H···*A*	*D*···*A*	*D*—H···*A*
C15—H15A···Cg1^i^	0.93	2.91	3.6318 (17)	138
C22—H22A···Cg2^i^	0.93	2.89	3.6438 (18)	140
C2—H2A···Cg3^ii^	0.93	2.74	3.4884 (18)	138

Symmetry codes: (i) *x*+3/2, −*y*+1/2, −*z*; (ii) *x*, −*y*−1, *z*−1/2.
